# Endometrial Cells Acutely Exposed to Phthalates In Vitro Do Not Phenocopy Endometriosis

**DOI:** 10.3390/ijms231911041

**Published:** 2022-09-20

**Authors:** Roberto Gonzalez-Martin, Andrea Palomar, Yassmin Medina-Laver, Alicia Quiñonero, Francisco Domínguez

**Affiliations:** Reproductive Medicine Research Group, IVI Foundation-IIS La Fe Health Research, 46026 Valencia, Spain

**Keywords:** di-(2-ethylhexyl)-phthalate, endocrine disrupting chemicals, endometriosis, endometrial stromal cells, endometrial epithelial cells

## Abstract

Environmental factors that have been linked to an increased endometriosis risk include exposure to di-(2-ethylhexyl)-phthalate (DEHP), an endocrine disruptor. This study aims to investigate whether DEHP in vitro exposure in primary endometrial stromal cells (EnSC), primary endometrial epithelial cells (EnEC), and the human endometrial adenocarcinoma cell line Ishikawa properly mimics alterations described in the eutopic endometrium of women with endometriosis. Primary EnSC and EnEC, isolated from six fertile egg donors, and Ishikawa cells were exposed to DEHP (0.1, 1, and 10 µM) and were assessed for viability, endometriosis markers (IL-6, VEGF-A, HOXA10, EZH2, and LSD1), steroid receptor gene expressions (ER-1, ER-2, PR-T, PR-B, and PGRMC1), and invasive capacity. Viability after 72 h of DEHP exposure was not significantly affected. None of the endometriosis markers studied were altered after acute DEHP exposure, nor was the expression of steroid receptors. The invasive capacity of EnSC was significantly increased after 10 µM of DEHP exposure. In conclusion, acute DEHP exposure in primary endometrial cells does not fully phenocopy the changes in the viability, expression of markers, or steroidal receptors described in endometriosis. However, the significant increase in EnSC invasiveness observed after DEHP exposure could be a link between DEHP exposure and increased endometriosis likelihood.

## 1. Introduction

Endometriosis is a common pelvic pathology associated with pain and infertility, with an estimated 10% of women worldwide experiencing the condition during their reproductive years [1]. Endometriosis is a complex, chronic, and systemic disease, and is characterized by the presence of endometrial tissue outside the uterine cavity, as well as cellular and molecular abnormalities shared in both eutopic and ectopic cells [1]. These abnormalities involve a state of progesterone resistance and estrogen dominance, a proinflammatory state, apoptosis resistance, increased angiogenesis and invasiveness, epigenetic defects at the level of DNA methylation, histone modifications, and ncRNA expression [1,2]. According to the theory of retrograde menstruation, eutopic endometrial cells with these abnormalities, through retrograde menstruation, establish themselves in the ectopic niches and evade cellular clearance [2].

The role of environmental factors in the pathogenesis of endometriosis has become increasingly apparent. Endocrine disrupting chemicals (EDCs) interfere with hormonal and immunological signaling and alter the epigenetics of target cells [3,4]. Specifically, a link between phthalate exposure and the risk of endometriosis has been described in several population-based case-control studies [5].

Phthalates are a family of organic chemicals commonly employed as plasticizers for the manufacture of polyvinyl chloride (PVC) plastics. Given the widespread use of plastic and the high absorption rate of phthalates, humans are exposed to high quantities of phthalates through food, water and other beverages, medications and nutritional supplements, medical devices, dental materials, cosmetics and perfumes, clothing, and toys, among others [4]. Many phthalates have been described as endocrine disruptors with both estrogenic and antiandrogenic effects [6,7].

Among phthalates, di-(2-ethylhexyl)-phthalate (DEHP) is the most widespread and is the most studied [8]. In in vivo studies with female murine models, it has been described that exposure to DEHP perturbs endometrial function through its interaction with hormonal receptors, which results in increased uterine glands and endometrial stromal cell proliferation [6], and impaired endometrial receptivity, which induces a lower implantation rate [9].

In the case of endometriosis, a significant increase in the risk of develop endometriosis has been estimated in women with higher DEHP exposure (OR = 1.42; 95% CI: 1.19–1.70) [10]. In addition, in other studies, higher levels of both the parental compound and its metabolites (MEHP, MEHHP, and MEOHP) have been detected in the plasma, serum, peritoneal fluid, and urine of women with endometriosis compared with healthy controls [5,11,12,13,14,15,16]. One of the mechanisms that could explain the relationship between DEHP exposure and an increased risk of endometriosis could be the induction of the above-mentioned molecular abnormalities in the eutopic endometrium of women with endometriosis.

Several studies have reported cellular aberrations in endometrial cells obtained from both disease free women and those suffering from endometriosis after acute DEHP exposure [16,17,18,19,20]. Acute DEHP exposure was found to increase cell viability, oxidative stress, and invasive capacity, altering the inflammatory response and steroid signaling in endometrial cells; these abnormalities are shared with both eutopic and ectopic endometrial cells in endometriosis [16,17,18,19,20]. In addition, in mouse models of endometriosis, exposure to DEHP seemed to increase the size and the proliferation of the ectopic endometrial implants [16]. Given these observations, acute DEHP exposure in endometrial cells may be useful for creating a model of endometriosis pathogenesis.

Because obtaining tissue samples from endometriosis patients is difficult, we aimed to generate a model of environmentally induced endometriosis via acute exposure to DEHP. We hypothesize that exposure to DEHP could induce alterations in endometrial cells similar to those described in the eutopic and ectopic endometrial tissue of women with endometriosis, which would serve as a tool to study the onset of this pathology.

Using primary endometrial stromal cells (EnSC), primary endometrial epithelial cells (EnEC), and the human endometrial adenocarcinoma cell line Ishikawa (as an established endometrial cell line serving as control between different groups), we assessed the cells’ ability to replicate the phenotypic alterations observed in the eutopic endometrium of women with endometriosis.

## 2. Results

### 2.1. Acute DEHP Exposure Did Not Affect the Viability of Endometrial Cells

**Figure 1:**
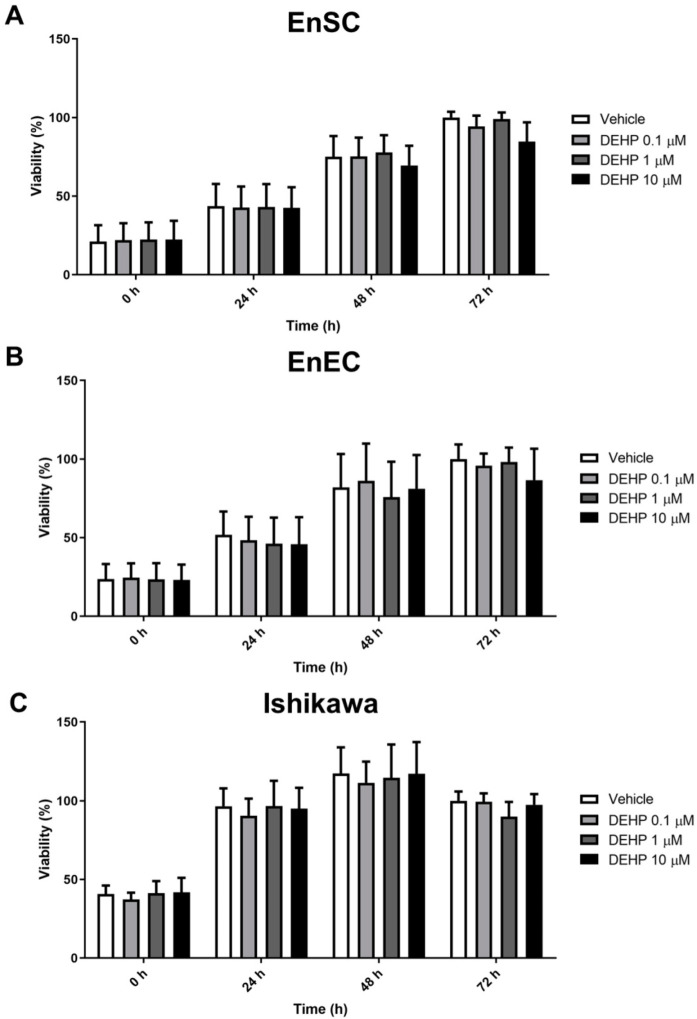
Endometrial cell viability following acute DEHP exposure. Primary EnSC (**A**), primary EnEC (**B**), and Ishikawa cells (**C**) were exposed to DEHP at 0.1, 1, and 10 µM. After treatment, viability was assessed with the MTS assay every 24 h. The results expressed in OD are relative to the vehicle at 72 h, represented as percentage ± SD of six different samples in the case of primary cells and three independent experiments in the case of Ishikawa cells. Each sample/experiment was performed in triplicate.

To determine the effects of in vitro DEHP exposure on the viability of primary endometrial cells (EnSC and EnEC, *n* = 6) and the Ishikawa cell line (*n* = 3), a time–dose MTS assay was performed. DEHP did not affect the viability of primary EnSC or EnEC at doses of 0.1, 1, or 10 µM at 24, 48, or 72 h (Figure 1A,B).

Ishikawa cells exposed to DEHP at the same doses did not change their viability at any of the times studied either (Figure 1C).

### 2.2. Endometriosis Marker Expression Was Not Altered after Acute DEHP Exposure

To check if in vitro DEHP acute exposure induces aberrations similar to those described in the eutopic endometrium of women with endometriosis, the gene expression of selected endometriosis markers was measured in primary EnSC, primary EnEC, and the Ishikawa cell line after vehicle or 0.1, 1, or 10 µM of DEHP exposure for 48 h.

**Figure 2:**
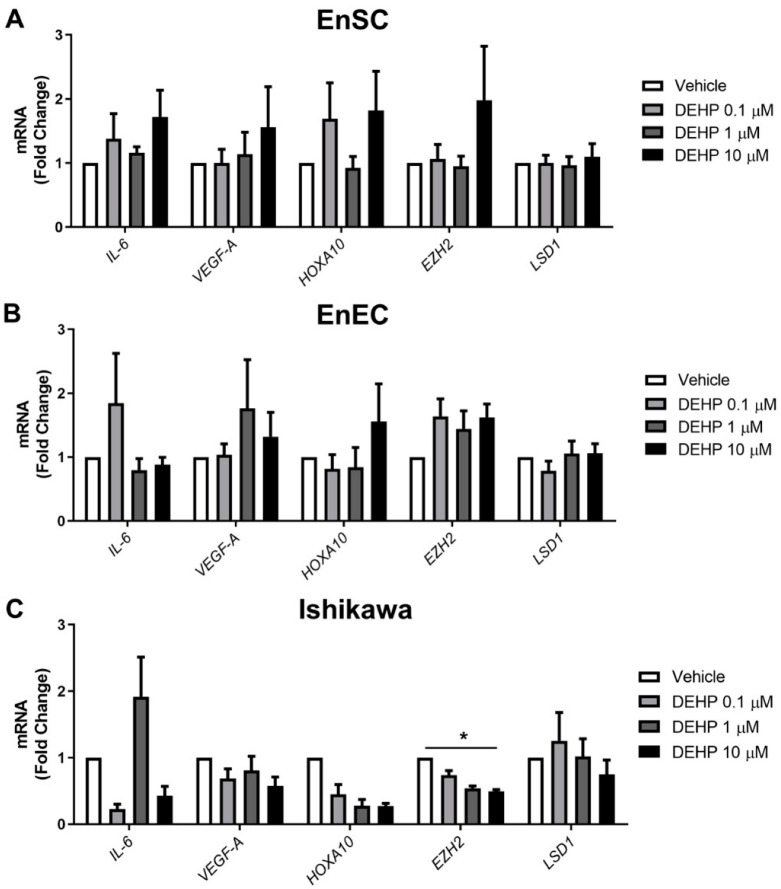
Endometriosis marker expression after acute DEHP exposure. After acute DEHP exposure in primary EnSC (**A**), primary EnEC (**B**), and Ishikawa cells (**C**); qPCR was performed for the endometriosis markers IL-6, VEGF-A, HOXA-10, EZH2, and LSD1. The results are represented as the mean ± SD of the data from six different samples in the case of the primary cells and three independent experiments in the case of the Ishikawa cells (* *p* < 0.05).

The gene expression of *IL-6* did not change for the primary EnSC and EnEC, nor for the Ishikawa cells after acute exposure to DEHP at the doses tested (Figure 2A–C).

The relative mRNA levels of VEGF-A were also not significantly altered in any cell types after acute DEHP exposure at the doses tested (Figure 2A–C). The expression of HOXA-10 was unaffected in the primary EnSC and primary EnEC after DEHP treatment (Figure 2A,B), but showed a trend toward a dose-dependent decrease in Ishikawa cells, although the changes were not significant (Figure 2C).

Acute DEHP exposure did not affect the expression of EZH2 or LSD1 in either the primary EnSC or primary EnEC at the doses tested (Figure 2A,B). However, the Ishikawa cells acutely exposed to DEHP showed a dose-dependent tendency to decrease their expression of *EZH2*, with fold changes related to the vehicle of 0.67 ± 0.05, 0.53 ± 0.08, and 0.47 ± 0.04 for DEHP of 0.1, 1, and 10 µM, respectively (*p* < 0.001). After Dunn’s multiple comparisons test, the difference was significant at the highest dose versus the vehicle (*p* < 0.05) (Figure 2C).

### 2.3. Acute DEHP Exposure Does Not Alter Steroid Receptor Expression in EnEC or Ishikawa Cells

To study the effect of acute DEHP exposure on steroid receptor signaling, primary EnEC (*n* = 6) and Ishikawa cells (*n* = 3) were exposed to the vehicle or to 0.1, 1, or 10 µM of DEHP for 48 h, and then the transcript levels of different steroid receptors (ER-1, ER-2, PR-T, PR-B, and PGRMC1) were measured using RT-q-PCR.

**Figure 3:**
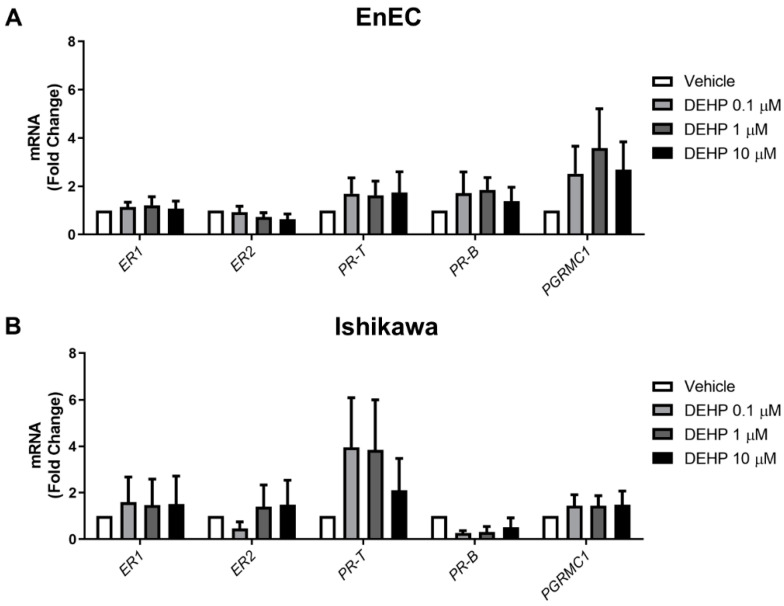
Steroid receptor mRNA in primary EnEC and Ishikawa cells following acute DEHP exposure. After acute DEHP exposure in the primary EnEC (**A**) and Ishikawa cells (**B**); qPCR was performed to measure the expressions of ER-1, ER-1, PR-T, PR-B, and PGRMC1. The results are shown as the mean ± SD for the data of six different samples in the case of EnEC and three independent experiments in the case of Ishikawa cells.

Overall, acute DEHP exposure did not significantly alter the expression of the steroid receptors in the primary EnEC or Ishikawa cells at the doses tested (Figure 3).

### 2.4. Acute DEHP Exposure Increases EnSC Invasiveness

**Figure 4:**
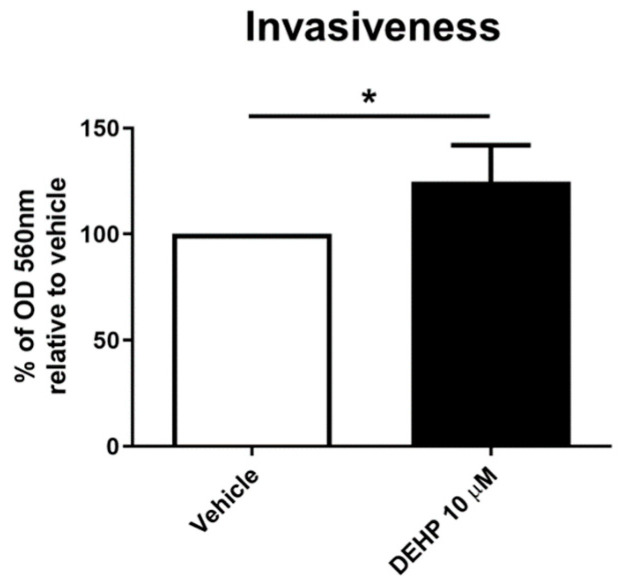
Cell invasiveness in primary EnSC following acute DEHP exposure. OD units are expressed as the percentage of migrating cells compared with the vehicle and represent the percentage ± SD for six different samples (* *p* < 0.05).

The invasiveness of EnSCs after 24 h of exposure to vehicle or 10 µM DEHP was assessed in vitro using a collagen invasiveness assay. The exposed EnSCs (*n* = 6) were harvested and immediately transferred onto the top of the collagen inserts. After 24 h, a significant increase of 24.72 ± 17.16% (*p* < 0.05) was observed in the number of cells exposed to 10 µM DEHP able to pass through the collagen insert to reach the chemoattractant stimulus as compared with the vehicle (Figure 4).

## 3. Discussion

The results obtained in this study suggest that acute in vitro DEHP exposure in primary EnSC, EnEC, and Ishikawa cells is not able to fully induce the molecular aberrations previously described in both the eutopic and ectopic endometrial cells of women with endometriosis. Our observations conflict with previous studies with an experimental design comparable to ours, with acute exposure (24–48 h) to DEHP doses in the picomolar to micromolar range. In these previous studies, the authors essentially observed an increase in the inflammatory response and invasiveness of the exposed cells [16,17,19,20,21], which we intended to replicate in our study. Although we were able to recapitulate the invasiveness increase after acute DEHP exposure, we were not able to induce the inflammatory, angiogenic, morphogenic, epigenetic, and steroid receptor expression alterations described in endometrial cells from women with this pathology. However, given the complex pathogenesis of endometriosis, in which numerous cell types are involved, our findings cannot exclude that DEHP does not act on other developmental stages or other cell types involved in its pathogenesis, facilitating the development of this disease.

Specifically, no statistically significant alterations in the viability of the three cell types were observed after in vitro DEHP exposure for 24–72 h. These results contradict those published by Kim et al., who described a significant increase in the viability of human EnSC and Ishikawa cells after 72 or 48 h, respectively, of DEHP exposure at 0.01 and 1 µM [17], respectively, and recently by Kim et al., who observed increases in the proliferation of Ishikawa, and endometriotic and unaffected endometrial epithelial lines 48 h after 25 μM of DEHP exposure [20]. However, our results are supported by the observations of Cho et al. and Huang et al. [18,19], who also did not observe alterations in the viability of endometrial cells after DEHP exposure during 24–72 h. Doses of 0.01, 0.1, and 1 nM and 0.2, 2, 20, and 200 µM were used in the studies of Cho et al. and Huang et al., respectively [18,19]. We employed doses of 0.1, 1, and 10 µM, which are comparable with the plasma exposure data of women with endometriosis, ranging from 1.28–6.24 µM [12,13,22], and are in the range of doses tested in the other similar studies [16,17,19,21], but our results do not support an effect from acute in vitro DEHP exposure.

As endometriosis has an important pro-inflammatory component [1], different inflammatory molecules have been studied as endometriosis markers after acute DEHP exposure (24–48 h). It has been reported that acute DEHP exposure, at doses ranging from picomolar to micromolar, during 24–48 h, increases NF-κβ signaling and the activation of downstream molecules, such as IL-1β, IL-8, COX-2, and NOS in EnSC [19,23] and IL-1β, IL-6, IL-8 TNF-α, INF-γ, MCP-1, RANTES, and COX-2 in EnEC [20]. Our results show that the expression of *IL-6*, a pro-inflammatory cytokine downstream of NF-κβ signaling, was unaffected after acute DEHP exposure, at doses ranging from 0.1–10 uM over 48 h, in both primary EnSC and EnEC and in Ishikawa cells, suggesting that acute phthalate exposure alone could not induce an inflammatory response in these cells.

The increase in angiogenesis observed in endometriosis allows cells to settle more easily in ectopic sites. We selected *VEGF-A* expression as an angiogenesis marker because its expression has been shown to be increased in the eutopic endometrium of women with endometriosis compared with healthy controls [24]. However, we observed no differences in the expression of *VEGF-A* after 48 h of DEHP exposure.

The expression of *HOXA-10*, an endometrial morphogenesis gene, is significantly decreased in women with endometriosis compared to controls [25]. However, we found no changes in the expression of this gene in primary endometrial cells acutely exposed to DEHP. In Ishikawa cells, there was a dose-dependent decreasing trend in *HOXA-10* expression, which could mean that these cells are slightly more sensitive to exposure to DEHP than the primary endometrial cells, and that damage may be beginning to occur at the level of endometrial morphogenesis. The same response was observed with the two epigenetic modification enzyme genes, *EZH2* and *LSD1*, whose expression was not altered after acute DEHP exposure in primary EnSC and EnEC cells, but the Ishikawa cells showed a tendency to decrease the expression of *EZH2* without alterations to *LSD1*. This is in contrast with what is described in the eutopic endometrium of women with endometriosis [26,27].

Epigenetic alterations are observed in endometriosis [28,29] and are linked to EDC exposure [30], so the fact that we did not observe alterations in the expression of two of the main epigenetic modulators in endometriosis [31] suggests that these epigenetic mechanisms are not being engaged in our model. DEHP exposure is mediated by its interaction with steroid hormone receptors [4], and in a previous study, an increase in *ER-1* and *PR* and a decrease in *ER-2* mRNA levels were observed in EnSC after DEHP treatment at picomolar range doses over 24 h [19]. Steroid receptor expression levels also differ between endometrial and endometrioid tissues and, in the case of PGRMC-1, the expression differs between normal endometrium and eutopic endometrium [32,33]. Our results showed no effect of DEHP exposure at doses of 0.1–10 µM over 48 h on the gene expression of steroid hormone receptors in either primary EnEC or Ishikawa cells.

Another EDC related alteration of EnSC in endometriosis is the increase in their invasive capacity. Matrix metalloproteinases (MMP) are markers of invasive capacity in endometriosis, with an increased expression of several of these proteases observed in tissue from women with endometriosis compared with healthy women [25,34,35]. An increase in MMP expression in endometrial cells has been reported after dioxin and DEHP exposure [16,18,36]. In the present study, we saw a significant increase in the cell invasion capacity of primary EnSC after acute exposure to 10 µM of DEHP. Kim et al. also reported an increase in invasiveness after DEHP exposure in EnSC and Ishikawa cells [16]. However, there were some methodological differences between that study and ours. Kim et al. used DEHP as a chemoattractant stimulus, while in our study it was employed as an invasion stimulator and FBS was used as a chemoattractant stimulus. We studied whether DEHP exposure could alter the function of the eutopic endometrium, transforming the cells to a more invasive state, while Kim et al. evaluated how the presence of DEHP at the destination site would attract cells.

Both the doses used and the exposure time were within those used in previous works evaluating the mechanism through which DEHP exposure might be related to the pathogenesis of endometriosis [16,17,18,19], and were meant to reflect human exposure [18]. However, this study had some limitations. First, we did not consider the levels of previous exposure of the participants to different EDCs, although we did use cells from the same participants as controls. Second, the endometrial biopsies were from healthy women undergoing COS, which could saturate the response to substances with a xenoestrogenic or endocrine disrupting effect. However, this kind of sample has been continuously used by our group and was shown to respond appropriately to the effects of other EDCs in previous work [37,38]. Third, it was not possible to include positive control cells from the eutopic endometrium of women with endometriosis to confirm the already described changes in this tissue; however, these alterations have been robustly described in the bibliography. Fourth, the sample number analyzed was limited, although it was similar to other studies [19]. However, although a larger number of samples could increase the statistical power of the observations, we did not find suggestive trends for the need for greater statistical power. Fifth, using large-scale techniques such as transcriptomics, epigenomics, and proteomics, other markers or pathways affected in endometrial cells by DEHP exposure may have been found. However, considering the functional data observed, other in vitro models could be more appropriate for this approach, as they are expected to be more representative of the alterations observed in endometriosis.

Our findings do not exclude the possibility that exposure to phthalates may increase the risk of endometriosis. In fact, the invasive capacity of the treated cells appeared to be increased; however, our objective was to establish an in vitro model that would allow us to phenocopy the main alterations reported in endometriosis, given the difficulties in obtaining these kinds of samples. However, the conditions of acute DEHP exposure were not sufficient to fully do so. Another model design may be necessary, potentially mimicking chronic exposure, or utilizing other cell types such as endometrial stem cells or samples from pre-pubertal stages of development, or by focusing on other cell types involved in the endometriosis pathogenesis, such as immune or peritoneal cells.

## 4. Materials and Methods

### 4.1. Sample Collection

Endometrial samples (*n* = 6) were obtained from healthy, fertile oocyte donors aged 18–33 years with regular menstrual cycles (21–35 days) and a body mass index (BMI) between 20 and 25 kg/m^2^. The donors were screened for endometriosis, pelvic inflammatory disease, uterine anatomic pathologies, and pregnancy three months prior to uterine biopsy. Biopsies were obtained with a Pipelle catheter (Laboratoire CCD, Paris, France) on the day of oocyte retrieval in controlled ovarian stimulation (COS) cycles and were processed to isolate endometrial stromal and epithelial cells.

Written informed consent was obtained from each participant (# 1612-FIVI-087-FD).

### 4.2. Isolation, Culture, and DEHP Exposure

Endometrial stromal cells (EnSC) and endometrial epithelial cells (EnEC) were obtained as previously described [39]. In brief, endometrial tissue was mechanically minced and then enzymatically disaggregated with a collagenase solution (0.1% (*v*/*v*) collagenase type IA; Sigma Aldrich, Madrid, Spain) in Dulbecco’s modified Eagle’s medium (DMEM; Sigma-Aldrich, Madrid, Spain) for 1.5 h at 37 °C.

The cell types were then separated by gravity sedimentation. For EnEC isolation, the sediment was treated with 200 µL of TrypLE Select Enzyme (1X) (Fisher Scientific, Madrid, Spain) for 1 min to finish the epithelial cell release, and then inactivated with 5 mL of DMEM and membrane filtered (100 µm cell filters, Celltrics, SYSMEX, Spain, Barcelona).

EnSCs were cultured in DMEM/F12 (with Phenol-Red) (Sigma-Aldrich, Spain) containing 0.1% (*v*/*v*) antibiotics. EnEC were cultured in DMEM/MCDB 105 (Sigma-Aldrich, Madrid, Spain) containing 0.1% (*v*/*v*) antibiotics, 2.2 mg/mL insulin from bovine pancreas (Milipore-Sigma, Madrid, Spain), and 10% (*v*/*v*) FBS. Serum-free media were used for the cell treatment in both cell types. The purity of cultures obtained was assessed according to the morphologic characteristics and was verified by immunofluorescence using vimentin, cytokeratin 18, and CD45 as markers for the stromal, epithelial, and immune cells compartments, respectively, as previously described [39,40] (Figure S1).

The human endometrial adenocarcinoma cell line Ishikawa was purchased from the European Collection of Authenticated Cell Cultures (Sigma-Aldrich, Madrid, Spain) and was cultured in MEM-Glutamax (with Phenol-Red) (Sigma-Aldrich, Madrid, Spain) containing 1% (*v*/*v*) non-essential amino acids, 2% (*v*/*v*) antibiotics, and 5% (*v*/*v*) FBS. Serum-free media was used for the cell treatments.

EnSCs, EnECs, and Ishikawa cells were exposed to DEHP (Sigma-Aldrich, Madrid, Spain) first diluted in dimethyl sulfoxide (DMSO; Sigma-Aldrich, Madrid, Spain) and then in serum-free medium (final DMSO concentration 0.002% (*v*/*v*)). The final DEHP concentrations were 0.1, 1, and 10 µM. These doses have been selected based on the DEHP levels detected in the plasma of women with endometriosis (1.28–6.24 µM) [12,13,22].

### 4.3. Cell Viability Assay (MTS)

The effects of DEHP on primary endometrial cells (EnSC and EnEC) (*n* = 6) and Ishikawa cell line (*n* = 3) viability were assessed using the MTS assay (Promega Biotech Ibérica, Madrid, Spain). EnSC or EnEC cells from each endometrial sample (2.0 × 10^4^ cells/well) were plated for each experiment in triplicate in 96-well plates and were allowed to attach overnight in 10% FBS medium. Thereafter, the cells were exposed to the vehicle (0.002% DMSO) or to 0.1, 1, or 10 µM DEHP in serum-free medium and were incubated at 37 °C and 5% CO_2_ in a humidified atmosphere for 0–72 h.

Subsequently, each 24 h, cell viability was assessed with the MTS reagent, following manufacturer’s protocol. The absorbance was measured on a microplate reader (spectraMAX 190, Molecular Devices, San Jose, CA, USA) at a wavelength of 490 nm. Optical density (OD) units were relativized to the vehicle at 72 h and the data were expressed in percentage ± standard deviation (±SD). Three replicates per condition were performed in each experiment.

### 4.4. Reverse Transcription-Polymerase Chain Reaction (RT-PCR) and Quantitative PCR (q-PCR)

Primary endometrial cells (EnSC and EnEC) (*n* = 6) and Ishikawa cells (*n* = 3) were exposed to the vehicle (0.002% DMSO) and DEHP (0.1, 1, or 10 µM), in serum-free medium, and were incubated at 37 °C and 5% CO_2_ in a humidified atmosphere for 48 h. The total RNA was isolated using an RNeasy Mini Kit (Qiagen Iberia, Madrid, Spain) and cDNA was generated from 0.5 µg of the total RNA using the PrimeScript™ RT reagent Kit (Takara Bio Europe SAS, France, Saint-Germain-en-Laye). The primers used for the amplification of each gene are shown in Table 1. Quantitative PCR was performed on a StepOnePlus Real-Time PCR System (Applied Biosystems, Madrid, Spain) with PowerUp SYBR Green Master Mix (Applied Biosystems, Madrid, Spain). Relative quantification of the mRNA expression was calculated using the 2^−ΔΔCt^ method, with GAPDH as an endogenous control. Data are presented as mean ± standard deviation (±SD) of the fold change relative to the cells exposed to the vehicle.

### 4.5. Invasion Assay

The effect of DEHP on the EnSC (*n* = 6) invasive capacity was assessed using a CytoSelect Cell Invasion Assay, following the manufacturer’s protocol (Cell Biolabs, CA, USA). Primary ESC grown to 60–80% confluence were starved in serum-free DMEM/F12 medium for 24 h. Then, the cells were resuspended at a density of 1 × 10^6^ cells/mL in a serum-free medium with either the vehicle (0.002% DMSO) or 10 µM DEHP, and were seeded on the top chamber. The bottom chamber was filled with 500 µL DMEM/F12 with 10% FBS as a pro-invasive stimuli. Absorbance was measured at a wavelength of 560 nm on a microplate reader (spectraMAX 190, Molecular Devices, San Diego, CA, USA). Optical density (OD) units were relativized to the vehicle and data were expressed as percentage ± standard deviation (±SD).

### 4.6. Statistical Analyses

Data from all of the experiments were examined using the Shapiro–Wilk test to assess whether they followed a normal distribution.

In the analysis of cell viability by MTS, the data followed a normal distribution, so they were analyzed by two-way ANOVA followed by a Bonferroni post-test to evaluate the impact of the concentration of DEHP used on the change in viability over the different exposure times. In the case of the Ishikawa cell line, given the low number of replicates, we employed a non-parametric test.

The mRNA expression level data did not follow a normal distribution, so the Kruskal–Wallis non-parametric analysis of variance method was applied, followed by Dunn’s Multiple Comparison test, to compare the effect of each DEHP dose on the fold change of the evaluated genes.

The invasiveness data also did not follow a normal distribution, so in this case, the non-parametric Wilcoxon signed-rank test was used to compare the invasiveness percentage of primary EnSC after the vehicle or 10 µM DEHP exposure.

Statistical computations were conducted using the GraphPad Prism 8.3.0 software (GraphPad Software, San Diego, CA, USA). *p* < 0.05 was considered statistically significant.

## 5. Conclusions

Our observations suggest that acute DEHP exposure in primary EnSC, EnEC, and Ishikawa cells does not fully trigger the phenotypic alterations described in the eutopic endometrium of women with endometriosis. We therefore do not consider it as a good model to study endometriosis in vitro. Nevertheless, we cannot exclude the possibility that such alterations might occur at later timepoints, following longer DEHP exposure, or in other cell types. Consequently, it cannot be concluded that exposure to phthalates does not affect endometrial physiology, or that it is not related to the appearance of endometriosis.

## Figures and Tables

**Table 1:** Primers used for RT-qPCR.

Gene	Forward Primer (5′-3′)	Reverse Primer (5′-3′)
*IL-6*	GATGGATGCTTCCAATCTGG	TGGCATTTGTGGTTGGGTCA
*VEGF-A*	AGGGCAGAGAATCACGAAG	TGGTGATGTTGGACTGCTCA
*HOXA10*	TGCTCCCTTCGCCAAATTA	GATGAGCGAGTCGACCAAA
*EZH2*	TTCATGCAACACCCAACACT	CTCCCTCCAAATGCTGGTAA
*LSD1*	CTAATGCCACACCTCTCTCAAC	CACACGAGTAGCCATTCCTTAC
*ER1*	GCTTCGATGATGGGCTTAC	CTGATCATGGAGGGTCAAATC
*ER2*	GATCGCTAGAACAACACACCTTAC	CGACCAGACTCCATAGTGATA
*PR-T*	GTGGGAGCTGTAAGGTCTTCTTTAA	AACGATGCAGTCATTTCTTCCA
*PR-B*	TCGGACACCTTGCCTGAAGT	CAGGGCCGAGGGAAGAGTAG
*PGRMC1*	GGAAGAGATGCATCCAGGG	TGAGTACACAGTGGGCTCCT
*GAPDH*	AGATCAAGAAGGTGGTGAAG	TTGTCATACCAGGAAATGAGC

## Data Availability

The data presented in this study are openly available in Mendeley Data at doi: 10.17632/864gyb3cvc.1.

## Supplementary Materials

The following supporting information can be downloaded at: https://www.mdpi.com/article/10.3390/ijms231911041/s1.
