# Supplementary material for: Endometrial Cells Acutely Exposed to Phthalates In Vitro Do Not Phenocopy Endometriosis
Source: Int J Mol Sci. 2022 Sep 20;23(19):11041. doi: 10.3390/ijms231911041 (PMC9569573; doi:10.3390/ijms231911041)
Supplement: Supplementary file 1 [file ijms-23-11041-s001.zip › ijms-1918877-supplementary.pdf]

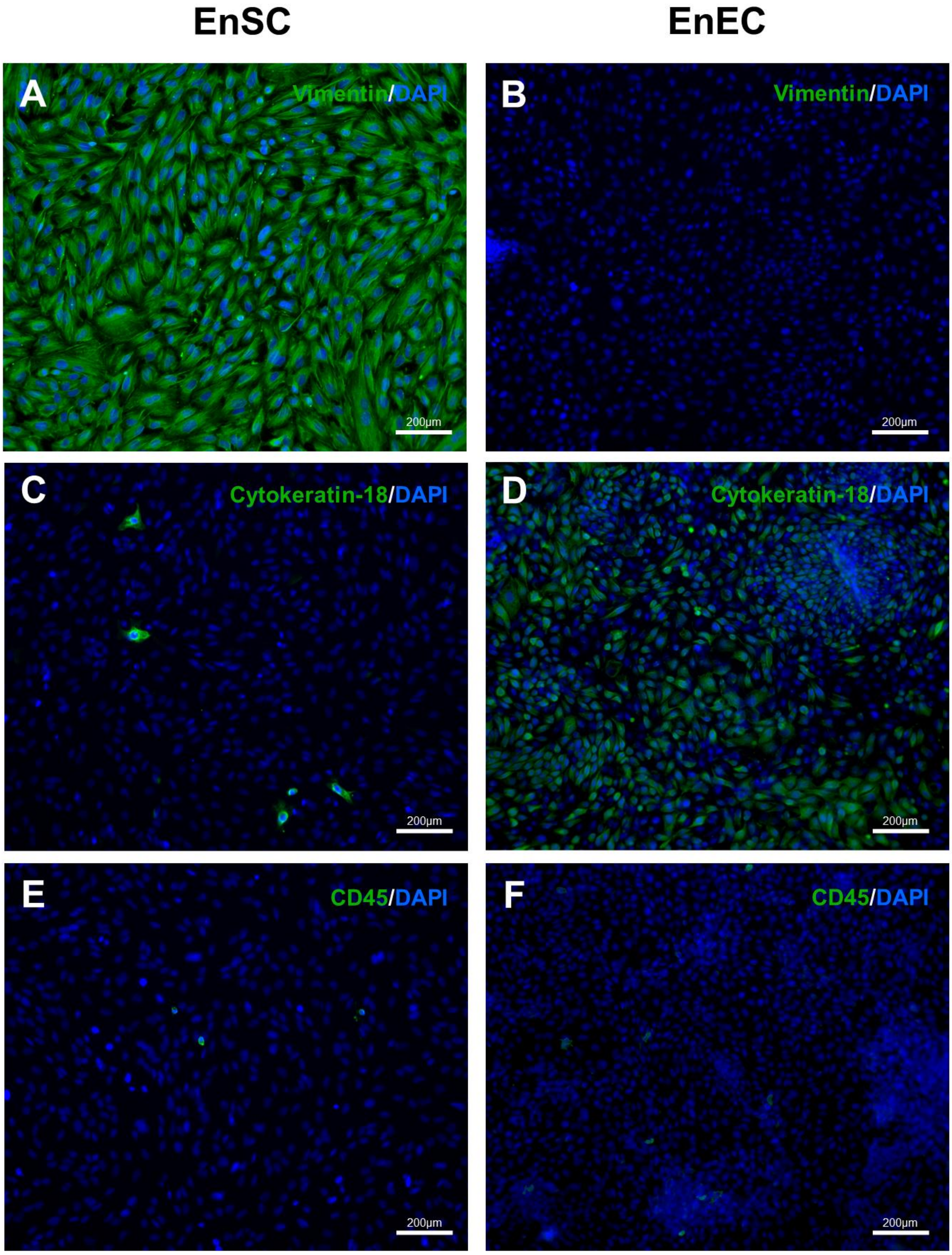

Figure S1. Primary EnSC and EnEC identification. Immunofluorescence for the stromal cell marker vimentin (A,B), epithelium cell marker cy-tokeratin-18 (C,D), and immune cell marker CD45 (E,F) of isolated EnSC (A,C,E) and EnEC (B,D,F) to demonstrate its purity. The microphotographs show merging of the indicated marker with the nuclear marker DAPI. The scale bar is shown in the lower right corner of each picture, with a length equivalent to 200 μM.
